# Highest Plasma Phenylalanine Levels in (Very) Premature Infants on Intravenous Feeding; A Need for Concern

**DOI:** 10.1371/journal.pone.0138532

**Published:** 2015-09-21

**Authors:** Ernesto Cortés-Castell, Pablo Sánchez-González, Antonio Palazón-Bru, Vicente Bosch-Giménez, Herminia Manero-Soler, Mercedes Juste-Ruiz, María Mercedes Rizo-Baeza, Vicente Francisco Gil-Guillén

**Affiliations:** 1 Department of Pharmacology, Paediatrics and Organic Chemistry, Miguel Hernández University, San Juan de Alicante, Spain; 2 Clinical Analysis Department, Alicante General University Hospital, Alicante, Spain; 3 Department of Clinical Medicine, Miguel Hernández University, San Juan de Alicante, Spain; 4 Department of Surgery, Paediatrics, Obstetrics and Gynaecology, University of Murcia, Murcia, Spain; 5 Department of Nursing, University of Alicante, San Vicente del Raspeig, Spain; TNO, NETHERLANDS

## Abstract

**Objective:**

To analyse the association in newborns between blood levels of phenylalanine and feeding method and gestational age.

**Study Design:**

This observational, cross-sectional study included a sample of 11,829 infants between 2008 and 2013 in a Spanish region. Data were recorded on phenylalanine values, feeding method [breast, formula, mixed (breast plus formula), or partial or fully intravenous feeding], gestational age in weeks (<32, 32–37, ≥37), gender and days since birth at the moment of blood collection. Outcomes were [phenylalanine] and [phenylalanine] ≥95th percentile. Associations were analysed using multivariate models [linear (means difference) and logistic regression (adjusted odds ratios)].

**Results:**

Higher phenylalanine values were associated with lower gestational age (p<0.001) and with intravenous feeding (p<0.001).

**Conclusion:**

The degree of prematurity and intravenous feeding influenced the plasma concentration of phenylalanine in the newborn. Caution should be taken in [phenylalanine] for newborns with intravenous feeding, monitoring them carefully. Very preterm infants given the recommended amount of amino acids should also be strictly monitored. These findings should be taken into consideration and call for adapting the amounts to the needs of the infant.

## Introduction

At birth once the umbilical cord is severed, the availability of amino acids (AA) for protein synthesis is suspended [1]. As soon as the infant begins breastfeeding (natural nutrition), the absorbed AA increase their plasma level and the high rate of protein turnover and synthesis required for growth [2].

Term and preterm infants fed breast milk or formula with different protein concentrations experience an increase in the total concentration of AA, depending on protein concentration received [3,4]. Plasma levels of essential AA are also higher in formula-fed infants compared to breast-fed infants [3].

In healthy individuals the intra-individual coefficient of variation for essential AA is lower than for non-essential AA, suggesting a tighter homeostatic control for essential AA. Specifically, the average concentration of phenylalanine (Phe) in plasma is 63 μmol/L, with an intra-individual coefficient of variation of 9.5% [5], with the variation in AA ranging from 12–32% [4].

Most infant formulas have a protein content of about 2.2 g / 100 kcal (1.5 g/dL), higher than that of breast milk (0.85 g/dL), so that protein intake in formula-fed infants is greater than that in breast-fed infants [3], with a resulting increase and difference in the pattern of AA [6]. Plasma levels of Phe, therefore, are also higher in formula than in breast milk [7]. Phe and Thyroxine (Tyr) levels are up to 20% higher among formula-fed versus breast-fed infants [8]. These increases may be even higher in preterm infants, especially very preterm infants because of possible metabolic immaturity and the use of formulas with increased protein to allow extra weight gain to ensure normal growth [9,10], along with decreased catabolism of essential AA caused by formulas rich in casein, as seen for Phe and Tyr [7]. Increases in nutritional AA in the preterm infant are currently under discussion, as the growth of infants breast-fed during the early stages is slower. Nevertheless, in the long term these infants experience fewer complications and a better development [11,12].

Neonatal screening was initiated with the study of phenylketonuria, resulting in improved outcomes in association with a Phe-controlled diet. The concentration of Phe in blood of healthy newborns is 50–110 μmol/L, while children with phenylalanine hydroxylase deficiency show hyperphenylalaninemia of varying severity, with levels persistently above 120 μmol/L [13]. In Spain, the cutoff established for the detection of hyperphenylalaninemia and phenylketonuria is 150 μmol/L [14]. The correct interpretation of changes in AA levels is therefore essential for prognosis, diagnosis and clinical monitoring of congenital metabolic disorders, including hyperphenylalaninemia.

Although much has been published on the adaptation of diet to gestational age, not much exists on the evaluation of the diet, gestational age and AA levels in the blood. Only extremely high values in newborns with phenylketonuria or hyperphenylalaninemia fed parenteral nutrition have been described [15]. Accordingly, we undertook a study to analyse this association. Our hypothesis was that Phe values vary according to neonatal gestational age and feeding method. We tested this hypothesis by calculating the expected values of Phe according to feeding method and gestational age.

## Materials and Methods

### Study population

The study population comprised preterm infants born in the province of Alicante (Spain). During the five-year study period, this region had about 16,000–18,000 births per year, of which 6.75% were preterm (<37 weeks) [16].

### Study design and participants

We undertook an observational cross-sectional study between May 2008 and June 2013, selecting a sample of newborns in the province of Alicante. The sample consisted of all preterm infants during this whole period and term infants born during the first six months of the study period. Inclusion criteria also required: 1) a registration of Phe with dried blood samples obtained on CF12 paper for neonatal screening for phenylketonuria and hyperphenylalaninemia; 2) informed consent for neonatal screening; 3) correctly completed record for the newborn and blood sample in accordance with preanalytical quality criteria [17]; 4) Phe values below 150 μmol/L (cutoff) [14]; and 5) the blood sample had to have been obtained within 14 days of birth (the reference timeframe for neonatal screening) [18].

### Variables and measurements

The primary outcome variable was the concentration of Phe (μmol/L). This was obtained through dried blood on paper with the fluorimetric method using the Neonatal Phenylalanine (Perkin Elmer) kit (7.8% monthly inter-assay coefficient of variation, internal control near cutoff). This variable was also assessed qualitatively: values in one group ≥95th percentile and in the other <95th percentile. Secondary outcome variables, which were measured at the time of blood sample collection, included: days from birth to blood sample collection (>/≤ 7 days), gender, feeding method [breast (human milk), formula, mixed (breast and formula), and partial or fully intravenous feeding] [19], and gestational age group (<32, 32–37 and ≥ 37 weeks) [20]. All variables were obtained through data collected on forms used for neonatal screening.

### Sample Size

The sample size was 11,829 newborns. The objective was to test for independence between the Phe groups (95th percentile) and the 4 feeding method groups (Pearson X^2^). As the sample was collected prior to calculating the sample size, the power of this contrast was calculated. To obtain an approximation of this value, a random sample of 1000 newborns was selected. Using this value and assuming a significance level of 5%, the power of the test was close to 100% [21].

### Statistical analysis

The descriptive analysis was performed using absolute and relative frequencies (qualitative variables: gender, gestational age group, sample collection >7 days, and feeding method), as well as means and standard deviations (quantitative variables: Phe values). A multivariate linear regression model was implemented using the Phe concentration as the dependent variable and all the secondary variables as explanatory variables. The goodness-of-fit of the model was obtained by ANOVA and all the basic hypotheses were tested using graphical and/or statistical tests. In addition, a logistic regression model was implemented using the Phe concentration groups (≥95th percentile) defined above as the main variable and all the secondary variables as explanatory variables. Thus, the adjusted odds ratios (OR) were obtained for a Phe concentration at or above the 95th percentile. Goodness-of-fit was assessed using the likelihood ratio test and the predicted probabilities of the model for Phe ≥95th percentile were calculated. Finally, box plots with predicted probabilities and values of Phe were created to help interpret the results.

All analyses were performed with a significance of 5% and confidence intervals (CI) were calculated for each relevant parameter. The statistical package used was IBM SPSS Statistics v19.

### Ethical consideration

Data analysis of the neonatal screening programme was approved by the Ethics Committee of the Valencian Community, and informed written consent of the newborn’s parent or guardian was obtained, in compliance with current legislation in medical ethics. The study is included in the neonatal screening programme.

## Results

The final sample consisted of 11,829 newborns. The descriptive characteristics are shown in Table 1. The proportion of feeding method was: breast, 54.5%; mixed, 25.3%; formula, 17.5%; intravenous, 0.5%. The gestational age was <32 weeks in 4.1% of the children.

**Table 1:** Quantitative and qualitative analysis of phenylalanine values (μmol/L).

				Adj. OR for	
	Total	B for Phe values		Phe ≥ 95th percentile	
Variable	n = 11,829	(95% CI)	p-value	(95% CI)	p-value
Male gender	6,141(51.9)	0.43 (-0.41,1.27)	0.318	0.96 (0.81,1.14)	0.636
Gestational age (w):					
≥37	4,702(39.7)	0		1	
32–37	6,578(55.6)	11.70 (10.77,12.63)	<0.001	6.71 (4.99,9.03)	<0.001
<32	487(4.1)	22.87 (20.56,25.18)	<0.001	21.04 (14.43,30.69)	<0.001
Collection >7 d	632(5.3)	3.27 (1.39–5.14)	0.001	0.88 (0.64,1.20)	0.422
Feeding method:					
Breast	6,447(54.5)	0		1	
Mixed	2,998(25.3)	-0.04 (-1.10,1.03)	0.948	1.09 (0.89,1.33)	0.419
Formula	2,073(17.5)	-0.24 (-1.45,0.94)	0.690	1.22 (0.98,1.53)	0.074
Partial or fully	64(0.5)	12.93 (7.19,18.67)	<0.001	2.69 (1.48,4.90)	0.001
intravenous					

Abbreviations: Adj. OR, adjusted odds ratio; CI, confidence interval; w, weeks; d, days. Goodness-of-fit of the models: ANOVA, F = 135.26 p<0.001; likelihood ratio test, 430.73 p<0.001.

Quantitative multivariate analysis of Phe (Table 1) showed statistically significant (p<0.001) differences in gestational age groups, feeding method, and days to sample collection. Compared to the term infants, those with a gestational age <32 weeks had an average Phe concentration 22.87 μmol/L higher, and those with a gestational age between 32 and 37 weeks 11.70 μmol/L higher. The infants who required partial or fully intravenous feeding also had a higher average concentration of Phe (12.93 μmol/L). Finally, children whose blood sample was collected more than 7 days after birth also had a higher Phe concentration (mean of 3.27 μmol/L).

Qualitative analysis of the concentration of Phe (95th Percentile) (Table 1) showed that a high concentration of Phe was significantly (p<0.001) associated with a lower gestational age (w) (≥37→OR = 1; 32–37→OR = 6.71, 95% CI: 4.99–9.03, p<0.001; <32→OR = 21.04, 95% CI: 14.43–30.69) and with partial or fully intravenous feeding (OR = 2.69, 95% CI: 1.48–4.90).

**Fig 1:**
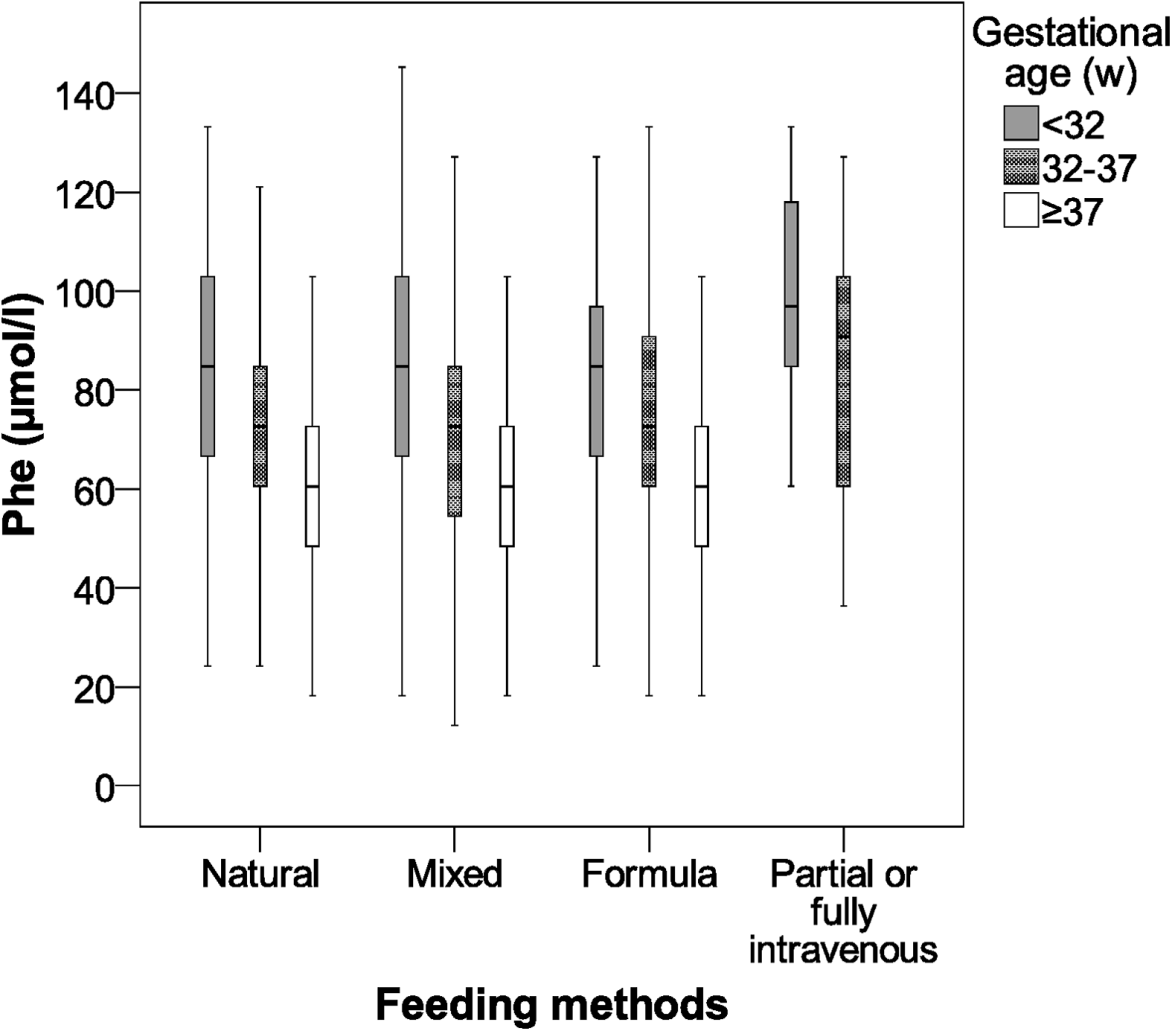
Phenylalanine values according to gestational age and feeding method in newborns. Phe, phenylalanine; w, weeks.

**Fig 2:**
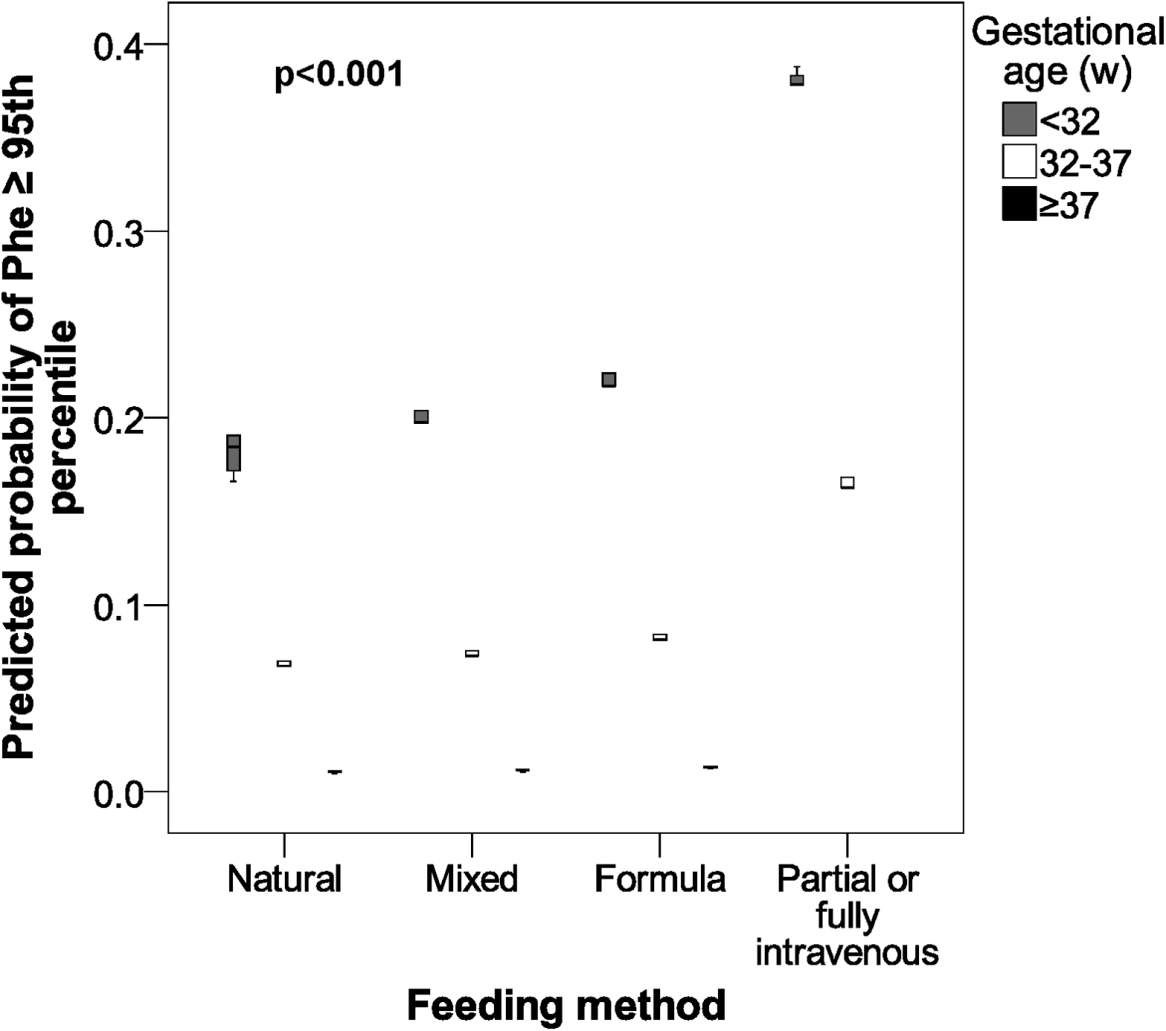
Predicted probability of atypical phenylalanine (≥95th Percentile) according to gestational age and feeding method in newborns. Phe, phenylalanine; w, weeks.

These results are shown in Figs 1 and 2.

## Discussion

This study shows differences in the Phe concentration in relation to gestational age and newborn feeding method. These differences existed at both mean values and high values (95th Percentile). For gestational age, the Phe concentration grew with the degree of prematurity. Furthermore, children who required partial or fully intravenous feeding showed the highest levels of Phe. These associations can be seen in Figs 1 and 2.

Our results show that Phe values did not depend on the feeding method, unless partial or fully intravenous feeding was required, in which case the Phe concentration was higher. This contradicts previous studies which found increases of up to 20% in plasma AA in formula-fed infants compared with breast-fed infants. However, these studies are older and not only have formulas evolved but the studies did not consider the influence of the degree of prematurity [3,7]. In our study, significantly higher values of Phe were observed in infants requiring partial or fully intravenous feeding compared to the others, probably because of the state of health of the newborn, which required the introduction of parenteral nutrition, which in turn contains greatly increased nutrients, and proteins in particular, in order to aid growth and weight gain [22]. This same situation has been reported in an infant with phenylketonuria who presented outrageously high levels with a parenteral diet [15]. The infants born after 32–37 weeks of gestational age, and especially those born at <32 weeks of gestation, tended to depend more on partial or fully intravenous feeding, due to their inherent anatomical and functional bowel immaturity and the high risk of necrotizing enterocolitis [10]. This feeding method is a way to provide a safe level of nutrient intake and to reduce morbidity and mortality, as well as ensure neurocognitive and psychomotor development similar to term children [23,24]. No uniform criteria exist for the amount of protein that partial or fully intravenous feeding should contain; the LSRO (Life Sciences Research Office) proposes that the protein intake range in preterm children should be between 3.4 and 4.3 g/kg/day [25]; the European Society of Paediatric Gastroenterology (ESPGHAN) recommends protein intakes depending on birth weight [26]. Specifically, the hospital setting for this study followed the recommendation of not exceeding 4.0 g/kg/day [11,27], in order to achieve a linear increase in weight gain in the newborn [24]. As these are established recommendations, similar to those for nasogastric feeding, we have to monitor strictly newborns on parenteral feeding,

The variations found in Phe concentration according to the gestational age group were highly significant, increasing as the gestational age decreased, with the highest values in very preterm infants. These results are congruent with those of other screening studies [28]. Furthermore, these infants very frequently present lung immaturity and complications such as neonatal respiratory distress syndrome, where elevated Phe levels have also been observed, probably due to decreased activity of phenylalanine hydroxylase caused by the depletion of its cofactor tetrahydrobiopterin [29].

Concerning the gender of the newborn, the findings showed no variation in plasma AA. However, the number of days between birth and sample collection was influential, with Phe levels being significantly higher in children who had the sample taken after the first week of life; this is in line with other studies [9,12].

This study shows that neonatal screening of aminoacidopathies using new technologies can be of great interest, increasing our understanding of the variability in AA values in blood and the possible modifying factors. Future application of these findings in the practice of infant nutrition may be interesting, given the large number of subjects involved.

### Strengths and limitations of the study

The main strength of this study is that it is the first (as far as we are aware) to analyse the differences in Phe concentrations in newborns according to the method of feeding and the gestational age, thus making the results novel. Moreover, the statistical power used with the sample was close to 100%, whereas most studies use values between 80% and 90% [21].

Regarding the limitations of the study, to minimize information bias, validated and calibrated equipment was used to obtain the Phe values, although this technology is now undergoing radical changes since the introduction of tandem-mass technology, which allows the quantification of virtually all AA. All other variables were obtained from the clinical record of the newborn, so we have to assume a possible information bias. Concerning selection bias, rather than taking a random sample of term infants, we used a determined period of time for collection. However, no variation was found in the average Phe values during the study period. Finally, we were not able to add the Phe levels of those infants who scored positively for the PKU screening as their data were not provided by the Ethics Committee. It would be interesting to study whether there is a legitimate concern for toxic Phe levels in our population e.g. by a follow up study.

## Conclusion

The degree of prematurity and intravenous feeding influenced the newborn plasma Phe concentration. Higher concentrations were not uncommon in very premature infants during intravenous feeding.

## Supporting Information

S1 Dataset Database with the study variables. (XLS)
